# Cohort profile: Women’s Healthy Ageing Project (WHAP) - a longitudinal prospective study of Australian women since 1990

**DOI:** 10.1186/s40695-016-0018-y

**Published:** 2016-10-04

**Authors:** Cassandra Szoeke, Melissa Coulson, Stephen Campbell, Lorraine Dennerstein, The WHAP Investigators

**Affiliations:** 1 Department of Medicine-Royal Melbourne Hospital, University of Melbourne, Parkville, Victoria 3050 Australia; 2 Melbourne Health, Melbourne, Australia

**Keywords:** Women’s health, Healthy ageing, Ageing, Menopause, Menopausal transition, Midlife cohort, Dementia

## Abstract

**Background:**

The cohort was commenced to examine women’s health from midlife (45–55 years) before the menopausal transition and into ageing.

**Methods:**

Randomised selection and assessment of 2,001 women living in the Melbourne metropolitan area was conducted by the Roy Morgan Centre in 1990/91. Of the 779 women who met the entry criteria for the longitudinal follow-up (aged 45–55 years, menstruating, having a uterus and at least one ovary and not taking hormone therapy) 438 agreed to be seen annually across the menopausal transition from 1992 to 1999. Longitudinal prospective follow-up since 2000 has continued intermittently (2002/03, 2004/05, 2012/13, 2014/15). Data collection has included fasting biomarkers in each year since 1992, clinical assessment, lifestyle and quality of life data, physical measures and validated questionnaire data. Participants have consented to data linkage and, to date, mammogram and BioGrid data have been accessed. Biobank storage including serum, deoxyribonucleic acid (DNA) storage and PAXgene tubes are maintained.

**Discussion:**

The WHAP has contributed to over 200 published research findings, several books, and book chapters in a variety of areas, including: health and wellbeing; mental and cognitive health; bone health; lifestyle, vascular risk and prevention; women’s health and hormonal transition; and cross-cultural research.

With all participants now aged over 70 years, the cohort is ideally placed to answer key questions of healthy ageing in women. With more than 25 years of longitudinal prospective follow-up this Australian dataset is unique in its duration, breadth and detail of measures including clinical review and specialized disease-specific testing and biomarkers. Ongoing follow-up into older ages for this long-running cohort will enable the association between mid to late-life factors and healthy ageing to be determined. This is particularly valuable for the examination of chronic diseases which have a 20–30 year prodrome and to provide knowledge on multiple morbidities. The dataset has a unique opportunity to improve our understanding of temporal relationships and the interactions between risk factors and comorbidities.

**Electronic supplementary material:**

The online version of this article (doi:10.1186/s40695-016-0018-y) contains supplementary material, which is available to authorized users.

## Background

The Women’s Healthy Ageing Project (WHAP) was initiated in 1990 to examine women’s health from midlife, before the menopausal transition, into ageing. The study is in a unique position to consider the life-course temporal changes in risk and the development of diseases of ageing, which due to the difficulty of maintaining longitudinal cohorts is an under-developed area of research. The driving vision of the scientific management advisory board and study leaders has been to use strategies outlined by the United States Centers for Disease Control and Prevention: 1) epidemiology to inform programs; 2) environmental approaches that promote health and support healthy behaviours; and 3) community resources linked to clinical services that sustain improvements in management [1].

The WHAP is unique in Australia as existing cohort studies from midlife (Australian Longitudinal Study of Women’s Health (ALSWH) [2], Melbourne Collaborative Cohort Study (MCCS), Household Incomes and Dynamics in Australia (HILDA)) do not have longitudinal assessments with physical measures, neuropsychological evaluation, lifestyle, and biomarker measures. Those with neuropsychiatric measures and biomarkers (Memory and Ageing Study (MAS) [3], Australian Longitudinal Study of Ageing (ALSA) [4], Australian Imaging, Biomarker and Lifestyle Study (AIBL) [5]) have only obtained information from those over 60 or 70 and the Path Through Life Study (PATH) [6] has no published information on neuropsychological function available before 60. Australian studies of ageing, like the Melbourne Longitudinal Studies of Healthy Ageing (MELSHA) [7] have only recruited after age 60. Few studies have detailed follow-up and biomarker measures and the only other study with amyloid imaging, AIBL, excluded individuals with vascular pathology, precluding the potential to understand this very influential factor contributing to morbidity in ageing [8].

As the large gains in health demonstrated in men from the 1960s to today are not matched in women [9], it is important to support women’s epidemiological research. Ninety percent of the gains in life expectancy for women are spent with a disability [10, 11], with women more likely to have a chronic illness, multiple disabilities [12], and higher health service use [13]. In addition there is an opportunity to address the significant lack of awareness of modifiable risk in women [14] and the absence of women-specific guidelines for risk modification, with most derived from studies in male-only cohorts [15]. The WHAP is committed to improving our understanding of risk factors and the accrual of disease over time, and moving towards improving overall health and wellbeing.

## Methods

### Study overview

The WHAP began in 1990, initially called the Melbourne Women’s Midlife Health Project (MWMHP). The study is unique in Australia and is a leader worldwide in being able to draw on an accumulated database incorporating biological, physical, and psychosocial measures of women’s experience from before the menopausal transition into subsequent decades [16].

The study has extensive validated measures of midlife health, lifestyle and biomarkers, including specialised measures of hormones and hormone exposures [17]. The outcome variables utilised in the study cover six major streams of research: Quality of Life and Ageing, Mental and Cognitive Health, Cardio - Vascular Health, Musculoskeletal and Bone Health, Lifestyle, and Women’s Health and Hormonal Transitions. The research governance structure of the WHAP includes oversight by a Scientific Advisory Board comprised of leading clinicians and academic researchers who cover a variety of specialty areas, as well as consumer representation [16].

The WHAP has demonstrated a successful model of longitudinal prospective research for more than two decades, attracting millions of dollars of philanthropic and peer-reviewed grant funding in addition to industry partnerships. It has published more than 200 peer-reviewed publications, review articles and books in women’s health, as well as contributing to websites, media and educational series on women’s health. The WHAP has formed international collaborations and held an important role in the development of international and national guidelines on women’s health.

### Sample selection and recruitment

To be eligible for the original study, participants had to be females aged between 45 and 55 years, and born in Australia [18]. Participants were recruited through the Roy Morgan Centre using randomly selected telephone numbers from a computerised database of the metropolitan Melbourne “White Pages” telephone directory. There were 54,078 calls made to households, with 9,329 of these calls (17.3 %) unanswered. Of the 44,749 calls placed to households, 40,364 (90.2 %) were terminated due to the household not having a woman available to complete the interview or the woman did not meet eligibility criteria (Australian-born women aged 45 to 55 years), and 1,447 (3.2 %) calls were terminated by the household before study eligibility was established. There were 2,938 (6.6 %) households called with a woman eligible to complete the interview; 105 (3.6 %) of the eligible women were unavailable due to being ill or absent during the study period. Of those available for the study (*n* = 2,833), 2,001 (70.6 %) women consented to take part in the baseline 2 hour telephone questionnaire, with 832 (29.4 %) women not agreeing to participate. Ethics approval for the original study was granted by the University of Melbourne Human Research Ethics Committee (Approval number: 585008_A_O_89_0489).

**Fig. 1:**
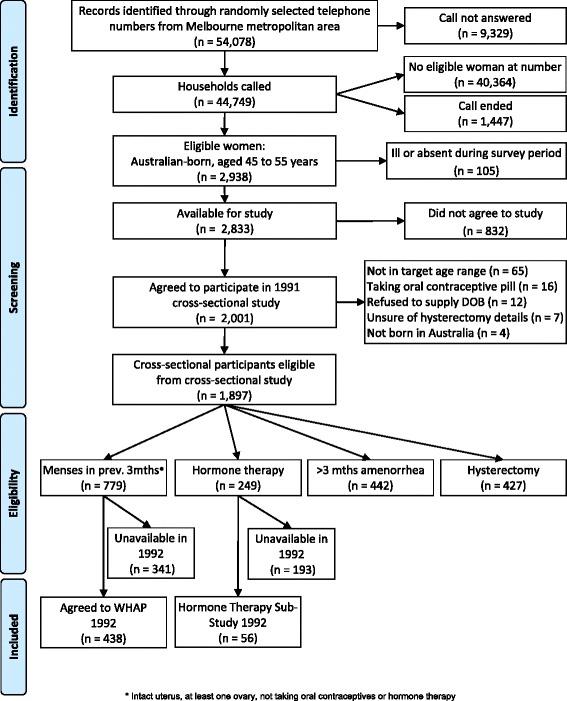
PRISMA Chart outlining the selection of the participants for the Women’s Healthy Ageing Project (WHAP) longitudinal study

**Fig. 2:**
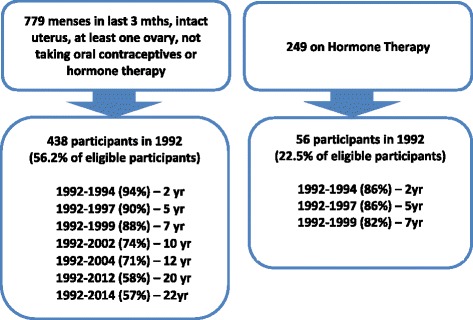
Cohort Longitudinal Follow-up and Retention

**Fig. 3:**
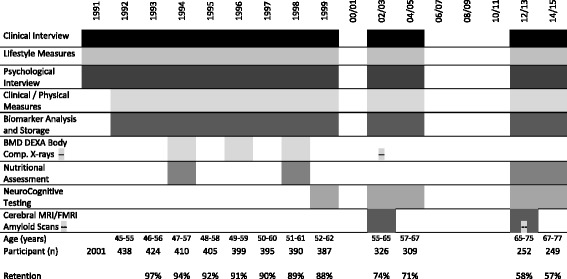
Participants retention and measures at each time-point across 25 years of follow-up 1990–2015

In 1992, the 779 eligible participants from the baseline phase of the study were invited to enroll in the follow-up phase of the study involving annual longitudinal follow-up. The inclusion criteria for this longitudinal study were women from the baseline study for whom menopausal status was able to be determined (had menses in the previous 3 months, had an intact uterus with at least one ovary, and were not taking oral contraceptives or hormone therapy). Figure 1 shows the acquisition for the original follow-up study. Of eligible participants a total of 438 (56.2 %) agreed to participate. An analysis of the characteristics of the women who agreed to participate in the follow-up study and those who did not showed that a greater proportion of those in the study reported that they had more than 12 years of education (34.3 vs. 24.3 %, *p* < .005), were in paid employment (71.4 vs. 63.0 %, *p* < .05), had perceived better health than most in comparison with women of the same age (48.5 vs. 38.1 %, *p* < .005), had exercised at least once a week (68.0 vs. 58.1 %, *p* < .005), had a Papanicolaou smear in the last year (58.3 vs. 50.0 %, *p* < .05), and had undergone dilatation and curettage (D&C; 46.5 vs. 38.4 %, *ρ* < .05) [19]. In contrast, women in both groups were similar in the following measures: age, body mass index (BMI), marital status, parity, symptoms, well-being, interpersonal stress, number of surgical procedures, proportion of current smokers, recent alcohol consumption, household composition, proportion who had breast checks in the last year, proportion who had a tubal ligation, use of medications, treatment for chronic conditions, and suffering from premenstrual complaints [19]. Retention of the original cohort across the initial follow-up period (1992 to 1999) was an impressive 88 % [17], by 10 years follow-up retention was 74 %, with more than 50 % follow-up in the 20th year [20] as shown in Figs. 2 and 3. Ethics approval for the WHAP longitudinal study was granted by the University of Melbourne Human Research Ethics Committee (approval numbers: 931149X (92–99), 010528 and 010411 (02–09), 1034765 and 1339373 (since 2012)). Those retained in the study were more likely to have higher levels of education and better health than those who were lost to follow - up over the course of the study [21].

### Procedure

The longitudinal follow-up of the WHAP were completed annually for the first eight follow-ups and intermittently thereafter (see Fig. 3), with all follow-ups completed in person. Participants were contacted by a researcher at each time-point to confirm their continuation with the study and to book a follow-up assessment time. Upon booking the assessment, participants were mailed the study information, participant consent form, and self-administered questionnaires before their assessment.

Participants were asked to fast overnight (minimum 10 hours) prior to their appointment. Upon arrival for assessment written informed consent was obtained and blood samples were taken for biomarkers, genetic testing, and biobank storage. Before the assessment commenced participants were served breakfast. The duration for the visit using the current protocol was approximately 4 hours [20]. All participant data was stored under unique identifier serial numbers and paper records of test results are kept securely. Figure 3 shows the included variables for all WHAP assessments.

### Data collection

#### Morbidity

Information regarding the participant’s age and their diagnosis of chronic medical conditions was obtained. Family history of chronic medical conditions was also obtained and a full list of medications recorded, in addition to any surgery or admission to hospital. Participants were specifically asked about 36 common diseases (including date of diagnosis and treatment provided) and 33 common symptoms (including severity scores). The Charlson Comorbidity Index [22] was calculated, predicting participants’ 1 year mortality risk.

#### Sociological and demographics

These are addressed in the demographics questionnaire and the core questionnaire. The questionnaires include items such as participant age, marital status, education, employment status, language, income, language, number of pregnancies, number of births, and number of children.

#### Quality of life

Items regarding social engagement, household composition, volunteer work, caring roles, and occupation were included in the core questionnaires. A wellbeing score was calculated from the Affectometer 2, a validated assessment of negative and positive mood as well as psychological wellbeing [23]. Self-reported health was measured using a brief questionnaire [24] at each follow-up. From 2012 onwards both the Short-Form Health Survey (SF-36) [25] (a 36-item health survey designed for use in the general population which addresses both physical and mental health) and the Activities of Daily Living Inventory (ADLI) [26] were completed. The ADLI consists of 23 items assessing changes in functional abilities of the participant, as reported by an informant such as the participant’s partner, child, or friend. The Short Form of the Informant Questionnaire on Cognitive Decline Evaluation (Short IQCODE) [27], included since 2012, also has items regarding participant function. Fatigue was assessed in each year with the Piper Fatigue Scale [28] included in 2014/15. Participants’ self-perceptions and attitudes to women in the workplace (1992, 1993), ageing [29] (1992, 1996), and menopause [30] (1992, 1993, 1997, 1998, 1999, 2002) were also measured. The Life Satisfaction Index scale (LSI-A) [31] and Life Satisfaction Index-Z scale (LSI-Z) [32] were administered in 1997. From 2004 onwards, questions regarding grandparenting (if they had grandchildren and how much time they spent minding their grandchildren) and if their now adult children had been demanding of them [33] were included.

Participants’ sexual functioning was assessed using the Personal Experiences Questionnaire (PEQ) [34] from 1992 to 1999, with the Personal Experiences Questionnaire - Short Form (SPEQ) [35] used from 2002 onward. The 12 items of the SPEQ were completed on a Likert scale, and relate to sexual experiences, enjoyment and thoughts, as well as the participant’s partner(s). The brief version of the Female Sexual Distress Scale (FSDS) [36] was used in 2002 and 2012 to measure participants’ personal distress related to sex and sexual functioning. Consisting of 12 items, the scale differentiates between women who are experiencing distress as a result of their sexual experiences and those who are not.

#### Experience of violence and abuse

Participants’ experience of violence was assessed in 1997 using a modified version of the Conflicts Tactics Scale (CTS) [37, 38]. The CTS asked participants about the behaviours used by their husband/partner in resolving conflict or arguments, with the modification to the scale being that participants were asked how frequently the 25 behaviours had occurred (never, once in the last year, more than once in the last year, anytime other than the last year). Participants were also asked about their experiences of both physical violence and sexual abuse before and since the age of 16 years. Participants completed the Vulnerability to Abuse Screening Scale (VASS) [39] from 2012 onwards, a 12-item scale designed to screen women for elder abuse.

#### Lifestyle and prevention

Participants were asked about their alcohol consumption (assessed as the number of standard drinks in the preceding 1 or 2 weeks) and smoking status (assessed as the number of cigarettes per day smoked, with calculated pack years) at each follow-up. Participants’ sleep was assessed via questions regarding the number of days they had trouble sleeping in the past 2 weeks and the severity of any problems. Attendance at national screening services (Papanicolaou smear, mammogram) was recorded, as well as personal preventative measures such as breast self-examinations. Mammography Imaging: The results of all mammograms completed by BreastScreen Victoria, a community breast screening programme, prior to 2002 were retrieved for consenting participants who did not have a history of breast cancer (68 % of the 387 participants in 1999) [40]. Original right-craniocaudal mammograms were examined; with the percent mammographic density (PMD) calculated using the total breast area on the mammogram and the area of dense tissue seen.

#### Physical activity and performance

Physical activity was recorded as the number of days in the last 2 weeks participants engaged in any physical activity and in physical activity that made them short of breath. In 1991 the types of physical activities that participants engaged in was also recorded, with the total energy expenditure per week calculated from this. Participants were asked in 2004 to complete a squat and a 10 metre jog. In 2012 the International Physical Activity Questionnaire (IPAQ) was added [41], which measures the number of days in the last 2 weeks and the number of days a month participants are engaged in physical activity for recreation or leisure. Since 2012 several measures of physical abilities were also included. The Timed Up and Go test [42] measures the time taken to rise from a seated position, walk 3 m from the chair, walk back to the chair and sit down again. Hand Grip Strength [43] was measured using a hand grip dynamometer. Participants were asked to squeeze the dynamometer as hard as they can, with the average result of three trials computed (in kilograms (kg)). In 2016 participants are asked to walk 5 metre wearing their regular shoes and in their regular pace, with the time taken to walk 4 metre recorded [44] as walking the greater distance avoids slowing down before reaching the 4 metre mark. Participants did not use a walking aid for the test unless they were unable to walk without using it.

#### Nutrition and diet

Each year participants were asked about intake of alcohol in the last two weeks and if they had experienced any loss of appetite, if they felt like not eating, or had any weight changes. The Food Frequency Questionnaire (FFQ) was used to assess participant nutrition in 1994, with the Cancer Council Victoria Food Frequency Questionnaire (CCFFQ) [45] used in 1998, 2012/13, 2014/15. The CCFFQ was developed specifically for use in Australian adults and is self-administered. It lists 74 food items grouped into four categories: cereal foods, sweets, and snacks; dairy products, meats and fish; fruit; and vegetables. A separate set of questions covers intake of alcoholic beverages. Participants nominate how often they consume the food items using a frequency response option ranging from ‘Never’ to ‘3 or more times per day’. It also includes three photographs of scaled portions for four foods (used to calculate a portion size calibrator), questions on the overall frequency of consumption of fruits and vegetables (used to calibrate the overestimation of these foods in the food list), and questions on consumption of foods such as bread that do not fit easily into the frequency format. Participants’ responses were used to calculate their Dietary Inflammation Index (DII) [46]. Nutrient intake is derived from participants’ FFQ responses, which is in turn used to calculate their DII. The DII predicts levels of inflammatory markers, and in turn, health outcomes influenced by chronic inflammation. Participants’ Mediterranean Diet Adherence score [47] and Western Diet score was also calculated, as well as their daily kilojoule, fat, protein, carbohydrate, sugars, and starch intake, and levels of dietary vitamin B12, folate, calcium, iron and other vitamins and minerals. Participants’ glycemic index and glycemic load were also calculated.

#### Menstruation and menopause

Participants were asked to report information regarding their menstrual periods until time of final menstrual period (FMP). In addition, questions specific to their menstrual history before the age of 40 years were included in 1994. Participants completed menstrual diaries and were asked questions to establish their menopausal status. Menopausal symptoms were measured using the checklist devised by Kaufert and Syrotuik [29, 48], with additional symptom items [49] included from 1994 onward. Extensive structured clinical interviews about hormone therapy (HT) use, indications, formulations, compliance and satisfaction were conducted.

#### Vascular health

Participants’ standing height (without shoes) was measured to the nearest 0.1centimetre (cm), with the highest of two measurements recorded. Weight was measured using regularly calibrated scales. Participants were weighed whilst wearing regular indoor clothing and no shoes, to the nearest 0.1 kg. Head, waist (at narrowest part of the trunk) and hip circumference were measured in cm. Triceps, biceps, subscapular, and suprailiac skin fold measurements were also taken from 1992 to 1999. Participants’ systolic blood pressure (SBP) and diastolic blood pressure (DBP) was measured utilizing a standardized blood pressure monitor. In addition to the morbidity screening detailed above, participants were asked about their experience of vascular conditions, significant symptoms, and family history of neurological, psychological, and vascular diseases and events. Cardiovascular risk scores were calculated [50–52]. Body Composition Imaging: Dual-Energy X-ray Absorptiometry (DEXA) scans were conducted at the Royal Melbourne Hospital up to 3 times over the period of the study (see Fig. 3). Both fat mass and lean tissue were measured as well as abdominal fat, with the measurement region for abdominal fat defined as “the area between the upper surface of the second lumbar vertebra, the lower surface of the fourth lumbar vertebra, and the lateral margins of the outer rib cage” [53].

#### Blood biomarkers

Following an overnight fast exceeding 10 hours, blood samples (80 ml) were collected by peripheral venous puncture into serum separator tubes. Within an hour of blood collection, the tubes were centrifuged, with 27 ml forwarded to a clinical pathology laboratory for immediate analysis. In addition, separate whole and fractioned blood samples from each participant were stored in liquid nitrogen in the biobank. The biobank consists of the remaining blood, which was fractionated into the following components: serum, plasma, platelets, red blood cell, white blood cell (in dH20) and white blood cell (in RNAlater, Ambion). These components were stored in liquid nitrogen in 92 aliquots (NUNC cryo-vials) which ranged in size from 0.25 ml to 1 ml. Stored blood samples were sourced from three different tube types: lithium-heparin tubes, EDTA tubes with added prostaglandin E1 (Sapphire Biosciences, 33.3 ng/ml), and serum tubes. Information on the assay techniques used across the project is included in Additional file 1. The blood biomarker analyses include:-

##### Hormonal assays

Follicle Stimulating Hormone (FSH) (international units (iu)/litre (l)), Estradiol (E) (picomole (pmol)/l), immunoreactive inhibin (unit (u)/l), Luteinising Hormone (LH) (iu/l), Testosterone (T) (nanomole (nmol)/l), Sex Hormone Binding Globulin (SHBG) (nmol/l), Free Testosterone Index (FTI), Dehydroepiandrosterone (DHEAS) (mmol/l), inhibinA (nanogram (ng)/l), inhibinB (ng/l) measured by radioimmunoassay and Thyroid Stimulating Hormone (TSH), Free Thyroxine (FT4), Free Triiodothyronine (FT3).

##### Lipid profile

Total cholesterol (millimole (mmol)/l), triglyceride (mmol/l), High Density Lipoprotein (HDL-C) (mmol/l) and Low Density Lipoprotein (LDL-C) (mmol/l).

##### Glucose sensitivity

Glucose, insulin (microunits (μunits)/milliliter (ml)). Inflammatory markers: C-Reactive Protein (CRP) (milligram (mg)/l), homocysteine (mmol/l). Vitamins: vitamin B12, vitamin D, serum folate, red cell folate.

Electrolytes and renal function: sodium, potassium, chloride, bicarbonate, anion gap, creatinine, Estimated Glomerular Filtration Rate (eGFR), urea. Liver function and proteins: Alanine Transaminase (AT), Alkaline Phosphatase (AP), Aspartate Transaminase (AST), Gamma Glutamyl Transferase (GGT), bilirubin, Albumin (Alb), Total Protein (TP). Metals: iron, ferritin, transferrin, ceruloplasmin, calcium, magnesium.

Full blood count, erythrocyte count, haematocrit, haemoglobin, platelet count, red cell count, leucocyte count, white cell differential, red cell differential, packed cell volume, Erythrocyte Sediment Rate (ESR).

##### Genotyping

Estrogen receptor polymoprhism, androgen receptor polmorphism, cytochrome 17 polymorphism, and apolipoprotein-E COMT, and Aromatase genotyping.

#### Musculoskeletal health

Participants were asked about their experiences of aches and joint pains, back pain, diagnosed arthritis or osteoporosis, and falls and fractures. Since 1999, a skeletal chart to note joints affected by pain and those with physician diagnosed arthritis has been utilized. Strength and balance testing was conducted in 2004/05. Quadriceps strength was measured using a seated force gauge test [54]. Participants sat in a chair (hips and knees in flexed position) with a force gauge attached to the base of the chair at the rear legs and a padded strap attached to one of the participant’s legs, slightly above their ankle. Participants were asked to extend the leg at the knee causing an isometric contraction of the quadriceps, with the quadriceps strength recorded as the maximum force exerted in kg. Balance was assessed using a variety of clinically recognised methods. The Step Test [55] was used to assess dynamic balance. Participants were asked to remove their shoes and stand with their feet parallel and 10 cm apart, with a 7 cm high block placed 5 cm in front of the participant. The test measured the number of times the participant could place their foot fully on the block and return it to the starting position (one cycle) during a 15 second (s) trial, with a trial completed for both feet. The participant’s score was the mean number of cycles completed from both trials. Static balance was assessed using a modified version of The Clinical Test of Sensory Interaction and Balance [56]. The test has four trials per leg, requiring the participant to stand on one leg for a maximum of 30 s, with the trials increasing in difficulty. For trial one, participants were asked to stand unassisted on a firm surface on one leg with their eyes open. This is repeated in trial two, but with participants closing their eyes during the trial. In trial three, participants were asked to stand unassisted on a high-density foam surface on one leg with their eyes open. In trial four, the participant repeats this with their eyes closed during the trial.

##### Imaging bone mineral density

Bone Mineral Density (BMD) was measured using DEXA at the Bone Densiometry Unit at the Royal Melbourne Hospital, using a Hologic QDR-1000 W densitometer in 1994, 1996 and 1998. Measures of BMD of the lumbar spine (the second to the fourth lumbar vertebrae), femoral neck and the proximal femur were taken. In addition to measures of BMD, body composition including abdominal fat and muscle mass were also obtained [57].

##### Hand and knee x-rays

In 2002/03 x-rays of both knees (antero-posterior view in full extension and skyline view in 45° flexion) and both hands were conducted along with C-telopeptide of type II collagen (CTX II) biomarker assays. The presence of osteophytes or joint narrowing evident in the x-rays in comparison to a published atlas of individual features [58] was used to categorise osteoarthritic disease using a four-point scale. Two trained investigators, who were blinded to any other participant information, independently scored the x-rays, with good intra- and inter-observer agreement [59]. Joint assessments were completed using validated measures. For hand x-rays, joints included in the classification are the distal interphalageal, proximal interphalageal, and the first carpo-metacarpal joint of the thumb [60]. For knee x-rays, the presence of osteophytes or joint narrowing was examined in the tibio-femoral and patella-femoral joints [61].

#### Mental health

Participants were asked to report their personal and family history of psychiatric illness, and medical history of depression, anxiety and other psychiatric illness. Each year mood was evaluated by semi-structured interview and the Affectometer 2, which provides a validated assessment of negative and positive mood as well as psychological wellbeing [23]. Depressive symptom measures were obtained from the Centre for Epidemiological Studies Depression Scale (CES-D) [62], administered since 2002. Individuals were required to rate their experience of 20 items on a 4-point Likert scale ranging from “rarely/none of the time” to “mostly/all the time” over the past week. In older populations somatic symptomatology can affect depression scales,therefore since 2012 we have included the Geriatric Depression Scale (GDS-15) [63]. Anxiety levels were obtained from the Hassles Questionnaire [64] which addresses everyday demands that may cause frustration and irritation, with the 86-item version used in 1992 and 1993, and the 23-item version used since 1994. In each year stress caused by demands, worry, illness and death was also recorded. The Hospital Anxiety and Depression Scale (HADS) [65], a 14-item assessment of anxiety and depression designed for use in those with physical health problems, has been included since 2012/13.

#### Cognitive health

The initial neuropsychological test of cognition, first completed in the 1998 follow-up was the Consortium to Establish a Registry for Alzheimer’s Disease (CERAD) [66] a 10-item supraspan word list score sensitive to early changes associated with dementia [67, 68]. From 2002 cognition was evaluated by a comprehensive neuropsychological evaluation, including measures of episodic memory, executive function, and speed of processing, and from 2012 CogState, a computerised cognitive testing scale [20], was introduced. The psychometric battery comprised several well-validated psychometric and neuropsychological tests and a computerized test of cognition since 2012. It includes tasks sensitive to the presence of mild cognitive impairment and cognitive decline. A clinical review panel reviewed all available medical, psychiatric, and neuropsychological information to confirm the cognitive health of individuals, and the panel was blinded to amyloid imaging results. Classifications were made consistent with current international criteria [14, 15].

The National Adult Reading Test (NART) [69] and the Wechsler Test of Adult Reading (WTAR) [70] were used to provide an estimate of premorbid intelligence quotient (IQ). The Mini-Mental State Exam (MMSE) [71] is a commonly used cognitive impairment assessment, indicating the participant’s level of orientation, attention, memory, language and praxis. The California Verbal Learning Task-II (CVLT-II) [72] and the CERAD Word List Recall Task [73] were used as measures of memory and learning. To evaluate working memory, the digit span tasks from the Wechsler Adult Intelligence Scale-III (WAIS-III) [74] were used. The Stroop interference task [75, 76] and Trail Making Test A and B [77] were used as measures of executive function. The Tower of London task [76] was used in 2002 and 2004 as a measure of executive function. The Controlled Oral Word Association Test (COWAT) [78] and the Boston Naming Test (BNT) [79] were included in the battery to evaluate language. The Digit Symbol task, a sub-scale of the WAIS-III [74], was used since 2012/13 to assess speed of processing. In 2002 and 2004, the letter-number sequencing task from the WAIS-III [74] and the Symbol Digits Modalities Test (SDMT) [80] were used as speed of processing tasks. The Logical Memory subtest (story A) of the Wechsler Memory Scale IV (WMS-IV) [81] was used to assess auditory memory from 2012, with the Faces task from the Wechsler Memory Scale III (WMS-III) [82] and the East Boston Memory Test [83] used in 2002 and 2004.

The spatial tasks in the battery were the Rey Complex Figure Test (RCFT) [84] and the Clock-Drawing Test (CDT) [76]. The Block Design Task and the Judgment of Line Orientation [74], both from the WAIS-III, were used as measures of visiospatial function prior to 2012. The Category Fluency task [85] assesses verbal executive function in participants. The computerised CogState battery [86] uses playing cards to test aspects of cognition. The Clinical Dementia Rating (CDR) [87] is a widely used clinical staging instrument for dementia, useful for globally staging the level of impairment based on a semi-structured interview. A detailed description of the neuropsychologic variables and the relationship between the different tests has been published [20].

In addition to the neuropsychological assessment, cognitive health was also evaluated using self-report and informant questionnaires. At each follow-up (FU) from 2002 participants were asked how their memory compared to others their age, and from 2012 were asked if they had experienced any problems with their memory in the previous seven days. From 2012, the Memory Complaint Questionnaire (MAC-Q) [88], a brief self-rating questionnaire regarding the participant’s current abilities compared to past abilities on shopping lists was also used. The Short IQCODE [27] is an assessment of subjective cognitive decline in the participant which is completed by an informant.

The Neuropsychiatric Inventory (NPI) [89] was used only for participants who had Alzheimer’s Disease (AD), and is administered to an informant. The CDR [87] was used to rate the severity of dementia symptoms, by assessing performance in personal care, home and hobbies, memory, orientation, judgment and problem-solving, and community affairs.

#### Cerebral imaging

A subset of 60 participants had 3 T magnetic resonance imaging (MRI; structural MRI and functional imaging) in 2002/03 and all participants of the WHAP were offered neuroimaging with 3 T MRI (structural MRI and functional imaging) plus Positron Emission Tomography (PET) with F-18 Florbetaben, a radioligand which enables quantification of AD pathology (β-amyloid) in vivo [90] in the 2012/13 follow up with 125 of 252 participants consenting. Over 50 % of participants had both PET and MRI compared with 12.5 % imaged in ADNI I [91] and 25 % imaged from the original AIBL cohort [5, 92]. All MRIs were performed on a Siemens 3 T Tim Trio Scanner. Five MRI sequences and functional magnetic resonance imaging (fMRI) are acquired: 1) T1 weighted Magnetization Prepared Rapid Gradient Echo (MPRAGE) MRI; isotropic 1 millimeteres (mm) voxel (Repetition time (TR) = 2300 milliseconds (ms), Echo time (TE) = 2.98 ms, flip angle = 9°); 2) 3D Fluid Attenuated Inversion Recovery (FLAIR), voxel dimension 0.90 × 0.97 × 0.97 mm (TR = 6000 ms,TE = 421 ms, flip angle = 120°, Inversion Time (TI) = 2100 ms); 3) Turbo Spin Echo T2W, 3 mm thickness, 0 mm gap, in plane resolution of 0.9 × 0.9 mm (TR = 3000 ms,TE = 101 ms); 4) Diffusion Tensor Imaging (DTI): Field-Of-View (FOV) 240, 55 slices, 2.5/0 thickness, TR 8600, TE 120, resolution 2.5 mm × 2.5 mm, B values 0 and 3000, 30 directions and bandwidth 1408; and 5) Arterial Spin Labelling (ASL): FOV 192, 14 slices, 6/1.5 thickness, TR 2500, TE 11, resolution 64, TI (1) = 700, TI (2) = 1800, bandwidth 2232 and 101 measurements. These sequences permit volumetric MRI analysis and evaluation of cerebrovascular pathology such as cerebral infarction and hyperintense white matter lesions, as well as the performance of atrophy-correction for PET data. fMRI, non-task related acquisition, was used for assessment of the default networks. Blood Oxygenation Level Dependent (BOLD) signal was collected continuously for 6 min. Participants were asked to keep eyes open and fixed on a projected cross hair. BOLD contrast, single-shot, T2*-weighted, gradient-Echo Planar Imaging (EPI) data was acquired continuously with an 8-channel birdcage radio frequency head coil. Each scan generated 180 volumes of whole-brain, 41-slice acquisition (TR = 3000 ms, TE = 30 ms, flip angle = 90°, voxel size = 3.0 mm^3^).

Resting state fMRI were conducted in 2002/03 and 2012/13 with T1-weighted images were acquired for anatomic localization, and a gradient ECHO EPI sequence was employed for fMRI studies (flip angle of 60°, TE 40 ms, 64by 64matrix, 24-cm FOV,TR = 1.5 s, 20 slices, slice thickness = 3.5 mm, skip = .5 mm). Event-related fMRI was conducted in 2002/03 with a 10 s interstimulus interval for activation tasks, with separate verbal and figural recognition memory tests, similar to the tasks used in previous PET studies [93, 94]. The memory tests involved verbal and figural encoding of abstract words and figures, yes/no recognition, and yes/no matching, the latter of which was used as a control task. In 2002/03 task oriented fMRI were acquired through the cerebral hemispheres in an oblique axial plane parallel to the anterior commissure - posterior commissure (AC-PC) line.. Each of the memory tests consisted of three separate tasks — encoding, yes/no recognition, and yes/no matching (control). Task order was: verbal encoding, figural encoding, 20-min delay concurrent with structural image acquisition, verbal recognition, figural recognition, verbal matching and figural matching. Test items were abstract words and figures. In each of the two encoding tasks, participants viewed 20 targets individually and pressed the ‘yes’ button on a hand-held unit each time an item appeared. In each of the two recognition tasks, 40 items were shown individually, and participants were instructed to discriminate studied from unstudied items by responding ‘yes’ or ‘no’. In each of the two matching tasks, 40 pairs of items were shown, and participants were instructed to discriminate identical from non-identical pairs.

F-18 florbetaben PET scans were conducted with participants receiving 250 MBq of F-18 florbetaben intravenously, with a 20 min acquisition commencing 90 min post injection. Standardized Uptake Values (SUV) were calculated for all brain regions examined and SUV ratios (SUVR) generated by normalising regional SUV using the cerebellar cortex. Neocortical SUVR, a global measure of β-amyloid burden, is expressed as the average SUVR of the area-weighted mean of frontal, superior parietal, lateral temporal, lateral occipital and anterior and posterior cingulate regions.

## Results

Whilst studies across the menopausal transition are prevalent in global research, there is very little information about the natural history of cardiovascular, bone and mental health functioning after the final menstrual period and how these are affected by a range of factors including premenopausal characteristics. This knowledge may allow the early identification of women who are at risk of disease, as well as clarifying the role of hormones and identifying modifiable factors amenable via interventions that could improve the health status of postmenopausal women in the future. There is also scant knowledge about changes in symptoms, mood, sexuality and other aspects of quality of life in the post-menopause. The main findings from each of the major research streams are briefly summarised below.

### Findings to date

#### Health and wellbeing

The first decade of the project focused on changes in health and symptoms related to menopause [95]. The association between hormonal changes and the menopausal transition and the relationship of these changes to symptoms [34, 49, 96] were an important contribution to women’s health. A novel aspect of the study was the contribution not only to health outcomes [97], but also self-rated health [24] and wellbeing [98–100]. The WHAP study addressed the paucity of information on positive mood [18, 101], life satisfaction [102] and attitudes to ageing [103] and their link to menopause [104]. These novel aspects of the study and the availability of baseline measures in middle age will provide important information on quality of life with ageing moving forward.

##### Sexual function

The study has led the research field in women’s sexual functioning [105–115], in particular with relevance to the changes over the menopause [116] and the influence of ageing [117, 118]. Risk factors for sexual dysfunction [119] have been identified and described for the late postmenopause [120]. The extensive research in this field has provided information on prevalence [121] and highlighted important considerations for study design [122] in measuring prevalence [123] and considering issues of reporting bias [124].

##### Violence in women

The study reported on the influence of violence on women’s mental [125] and overall health [126, 127]. It also reported the prevalence of violence [128] in mid-aged women. In the latest follow-ups we have included the VASS for a broader view of violence in older people.

#### Mental and cognitive health

A unique aspect of the WHAP is the focus on positive, not just negative, mood and the major factors which are associated with a positive mood across early ageing have been published [129]. The importance of lowered mood across a woman’s life [23, 129–131] and relevance to mental health treatments [132] and psychotropic drug use [23, 129–131, 133] was also reported.

The WHAP cognitive database is unique in the world with the availability of longitudinal prospective measures from 45 years of age (including blood and imaging biomarkers) combined with neuropsychiatric evaluations from participants in their 50s and the availability of late-life specialized neuro-imaging including fMRI and measurement of cerebral amyloidosis [20]. Earliest changes in cognitive decline from age 50 have been examined, and the importance of social engagement [33, 134–137] and minimizing vascular risk [138] to improve cognitive health have been reported. These findings are important in the context of the paucity of cognitive data in those under 60 in conjunction with recent research noting that the earliest changes of brain pathology associated with dementia can already be found in 50 year olds [139]. In addition the results of our early work have highlighted the importance of longitudinal studies in cognitive research as timing and duration of exposures is relevant to outcomes for blood pressure [140], lipids [141], hormones [142–146] and other modifiable risk factors [142]. Genomic associations with early cognitive decline have been examined with the influence of estrogen polymorphism published [147]. There is an absence of published normative data in Australia and the first published normative papers came from the WHAP data set for memory [148] and executive function [149].

#### Bone

Bone health in women is extremely important and the first decade of the study BMD over the menopausal transition [150], reported the bone loss in menopausal women [151] and also identified key risk factors for osteoporosis in Australian women [152]. There were several studies that reported hormonal influence on BMD [153, 154]. In our regular annual feedback, we have had consistent requests from the participants to have repeat BMD testing, and plans are currently underway to arrange this. This is not surprising given the high incidence of osteoporosis in older women and the importance of preventing bone loss in ageing. Future work will focus on the influence of lifespan exposures and importance of timing on changes in BMD.

The study was the first to highlight that the most common symptom of the transition was, in fact, “aches and joint pains” [155] rather than “hot flashes”. Further work explored these common symptoms and noted the prevalence of osteoarthritis in women [59], as well as its relationship to menopause [156] and risk factors [157]. This research also contributed to important study design considerations for epidemiological research into arthritis [158].

#### Lifestyle, vascular risk and prevention

The WHAP has reported on preventive testing with mammography [40]. Recent reviews have noted the paucity of research examining cardiac risk factors specific to women [159, 160]. The WHAP has shown that weight gain and obesity are prevalent [161] and that weight does influence hormone levels [53]. The earlier phase of the WHAP reported risk factors for coronary heart disease across the menopausal transition [162] and the influence of hormones [163, 164]. Newer measures of vascular risk have also been examined such as impaired fasting glucose [165], homocysteine, CRP and hormone therapy [166]. There have been a number of papers looking at midlife physical activity, the changes over menopausal transition [167] and impacts on health [168, 169]. These factors will be particularly important to examine in terms of longer term health outcomes, now being measured in follow-up.

Full nutrition surveys have been conducted over the 20-year study and some early work examining the relationship of diet to vascular risk [170] and consumption of dietary phytoestrogens [171] has been published. Alcohol use and its relationship to key variables has been reported for early ageing [172]. However the large and relevant nutrition data-set is still largely untapped.

#### Women’s health and hormonal transition

The study made major contributions to methodological considerations of reproductive ageing with a large body of published research towards defining the stages [173–178] and symptoms [179, 180] of the menopausal transition. Our study also combined with North American cohorts in the ReSTAGE collaboration to inform the new Stages of Reproductive Aging Workshop + 10 (STRAW + 10) criteria for postmenopause [181].

The study has provided much information on the prevalence and importance of symptoms [17, 182–184] and their influence on health-seeking behavior [185, 186]. The cohort has informed management of incontinence [187] and in particular vasomotor symptoms [188, 189] with important research on the relationship of these symptoms to the menstrual cycle [190].

Some of the most important work on defining the hormonal changes across the menopause came from the first decade of this study [19, 191–200]. Prior to these results, it was believed that androgens such as testosterone dropped significantly during the menopausal transition. Our study showed that there was little change in testosterone during this time period and the level of free or non-bound testosterone actually increased with the transition. The study also described patterns of hormone therapy use in Australian women [201, 202]. The impact of obstetric procedures such as tubal ligation, D&C [203] and hysterectomy [204], including longer term outcomes [205], was also examined.

#### International focus

The study has been active in international collaborative research including cross-cultural comparisons [206–213]. It has contributed to international standards for sexual function research [214, 215] and recommendations for staging reproductive ageing [181] including defining criteria [216].

There are several position papers on the menopausal transition [217, 218], vasomotor symptoms [219], somatic symptoms [220], and on hormonal change [217]. It has also contributed influential position papers regarding statistical techniques and study design for longitudinal data [221] in addition to clinical management position papers on mental health [211–213] and osteoporosis [152]. The research program has developed and validated scales for measuring sexuality [35, 222] and physical activity in midlife women [223].

## Discussion

The WHAP has evolved over the two decades from a small research team into a broad international network of researchers with oversight from the Scientific Advisory Board. The importance of robust ongoing measures across the lifespan continues to provide crucial evidence on the timing and duration of risk and potential therapeutic windows. This is particularly relevant for chronic diseases. The holistic approach taken by the research group in conjunction with our collaborative partnerships has seen validated scales from various specialty areas utilized which will now permit genuine multi-morbidity research as the women enter ageing.

### Strengths and limitations

The value of longitudinal research is in repeated measures, but particular challenges arise in a study of now more than two decades. Technological advances lead to the development of new and improved measures over time. In research strategic planning, considering the use of those cutting edge technologies, is an important consideration for “future-proofing” the study. Such an example is our choice to conduct 3 T MRI scans in 2002 (then only available in Melbourne, at the Brain Research Institute). A decade later in 2012, when 3 T scans were standard in all centres, comparable scans could be obtained. An important way to counteract biomarker technological advances is biobank storage, which has been a crucial component of our study, allowing novel biomarkers to be tested on stored samples. Some changes are difficult to anticipate, but our consenting for data-linkage in the early years of the study when only basic government registry data was available now facilitates national and international collaborations and linkages to widely utilized electronic data sources.

Another limitation of such longitudinal research is that validated scales from population-based research have generally not been validated in older adults (over 70 years). Further methodological challenges occur with the development of particular rating scales, such as the CES-D (validated to age 65) which has an alternate geriatric form, the GDS-15 (validated in older ages). This circumstance requires consideration of the value of consistent follow-up measures with the same scale versus the evidence that inclusion of somatic symptoms in older age groups leads to over-reporting of depressed mood. The narrow age range of the sample has allowed examination of factors and outcome without a large confounding from age, but is only then representative of a decade age span. As in any cohort of long duration, those participants remaining in the follow-up are also healthier and slightly more educated [20]. Initial inclusion for the longitudinal cohort excluded those with hysterectomy which impacts the generalizability of the findings.

### Future

The WHAP is continuing follow-up in order to provide an essential resource of longitudinal prospective research in women from 45 years of age through to their 90s, notably absent in Australia. Research areas will focus on the major causes of morbidity and mortality in postmenopausal women which include cardiovascular disease, mental health disorders (depression and dementia), and musculoskeletal and bone health. These have all been linked to menopausal hormonal changes. Large clinical trials of various forms of hormone therapy have shown age-dependent variation in results with increasing evidence of the timing hypothesis [224].

Chronic diseases are recognised as the largest contributor to death, morbidity and disability in our ageing society [225, 226] and globally in the western world [227]. The aetiology of these diseases involves decades of exposure, which therefore requires decades of longitudinal follow-up to examine factors associated with disease development. In addition, chronic diseases are known to have large overlaps in risk factors and 80 % of our elderly suffer from 2 or more morbidities [228]. However research is disease-centered with a recent Lancet publication noting that this practice has led to a lack of translation to practice given the majority of those over 50 years of age have not one but several coexisting conditions [229]. Our study, with measures across several disease areas, is ideally placed to examine such multi-morbidity outcomes. Therefore, the greatest value to this field of research will now come from follow-up measures as women age. The WHAP study will be uniquely placed to examine midlife associations with later cognitive, bone, mental and vascular health, cerebral, bone and blood pathology, and function and also to examine temporal aspects on the development of disease – a key factor in chronic diseases which have a 20–30 year prodrome.

## Collaboration

The WHAP encourages collaboration and in particular cross-cultural studies and combined cohort studies that leverage synergistic datasets. The data is available through BioGrid Australia Limited which is a leading data sharing organisation providing a secure infrastructure that advances health research by linking participant-level, privacy-protected and ethically approved, clinical, biospecimen, genetic and imaging data among a wide network of health collaborators [230]. The cognitive dataset is also included in the Global Alzheimer’s Association Interactive Network [231]. The research program has nurtured a growing cohort of alumni from undergraduate students to higher degree students, scientists and health professionals completing postgraduate research training. The data-set is open for analysis towards higher degree study [16].

## Additional file

Additional file 1: Changes to the instrumentation and assay techniques for blood biomarker analysis across two decades of the Women’s Healthy Ageing Project.ᅟ(DOCX 13 kb)

## Abbreviations

AC-PC: Anterior commissure - posterior commissure
AD: Alzheimer’s disease
ADLI: Activities of daily living inventory
AIBL: Australian Imaging, Biomarker and Lifestyle Study
Alb: Bilirubin albumin
ALSA: Australian Longitudinal Study of Ageing
ALSWH: Australian Longitudinal Study of Women’s Health
AP: Alkaline phosphatase
ASL: Arterial spin labelling
AST: Aspartate transaminase
AT: Alanine transaminase
BMD: Bone mineral density
BMI: Body mass index
BNT: Boston naming test
BOLD: Blood oxygenation level dependent
CCFFQ: Cancer council victoria food frequency questionnaire
CDR: Clinical dementia rating
CDT: Clock-drawing test
CERAD: Consortium to Establish a Registry for Alzheimer’s Disease
CES-D: Centre for Epidemiological Studies Depression Scale
cm: centimetre
COWAT: Controlled oral word association test
CRP: C-reactive protein
CTS: Conflicts tactics scale
CTX II: C-telopeptide of type II collagen
CVLT-II: California verbal learning task-II
D&C: Dilatation and curettage
DBP: diastolic blood pressure
DEXA: Dual-energy X-ray absorptiometry
DHEAS: Dehydroepiandrosterone
DII: Dietary inflammation index
DNA: Deoxyribonucleic acid
DTI: Diffusion tensor imaging
E: Estradiol
eGFR: Estimated glomerular filtration rate
EPI: Echo planar imaging
ESR: Erythrocyte sediment rate
FFQ: Food frequency questionnaire
FLAIR: Fluid attenuated inversion recovery
FMP: Final menstrual period
fMRI: Functional magnetic resonance imaging
FOV: Field-of-view
FSDS: Female sexual distress scale
FSH: Follicle stimulating hormone
FT3: Free triiodothyronine
FT4: Free thyroxine
FTI: Free testosterone index
FU: follow-up
GDS-15: Geriatric depression scale
GGT: Gamma glutamyl transferase
HADS: Hospital anxiety and depression scale
HDL-C: high density lipoprotein
HILDA: Household incomes and dynamics in Australia
HT: Hormone therapy
IL-1: Interleukin-1
IL-6: Interleukin-6
IPAQ: International physical activity questionnaire
IQ: Intelligence quotient
iu: international units
kg: kilograms
l: litre
LDL-C: Low density lipoprotein
LH: Luteinising hormone
LSI-A: Life satisfaction index scale
LSI-Z: Life satisfaction index-Z scale
MAC-Q: Memory complaint questionnaire
MAS: Memory and Ageing Study
MCCS: Melbourne Collaborative Cohort Study
MELSHA: Melbourne Longitudinal Studies of Healthy Ageing
mg: milligram
ml: milliliter
mm: millimeteres
mmol: millimole
MMSE: Mini-mental state exam
MPRAGE: Magnetization prepared rapid gradient echo
MRI: Magnetic resonance imaging
ms: milliseconds
munits: micro-units
MWMHP: Melbourne Women’s Midlife Health Project
NART: National adult reading test
ng: nanogram
nmol: nanomole
NPI: Neuropsychiatric inventory
PATH: Path through life study
PEQ: Personal experiences questionnaire
PET: Positron emission tomography
PMD: Percent mammographic density
pmol: picomole
RCFT: Rey complex figure test
s: second
SBP: Systolic blood pressure
SDMT: Symbol digits modalities test
SF-36: Short-form health survey
SHBG: Sex hormone binding globulin
Short IQCODE: Short Form of the informant questionnaire on cognitive decline evaluation
SPEQ: Personal experiences questionnaire - short form
STRAW + 10: Stages of reproductive aging workshop + 10
SUV: Standardized uptake values
SUVR: Standardized uptake value ratio
T: Testosterone
TE: Echo time
TI: inversion time
TP: Total protein
TR: Repetition time
TSH: Thyroid stimulating hormone
u: unit
VASS: Vulnerability to abuse screening scale
WAIS-III: Wechsler adult intelligence scale III
WHAP: Women’s healthy ageing project
WMS-III: Wechsler memory scale III
WMS-IV: Wechsler memory scale IV
WTAR: Wechsler test of adult reading
