# Supplementary material for: Cohort profile: Women’s Healthy Ageing Project (WHAP) - a longitudinal prospective study of Australian women since 1990
Source: Womens Midlife Health. 2016 Oct 4;2:5. doi: 10.1186/s40695-016-0018-y (PMC6300017; doi:10.1186/s40695-016-0018-y)
Supplement: Supplementary file 1 — Changes to the instrumentation and assay techniques for blood biomarker analysis across two decades of the Women’s Healthy Ageing Project.ᅟ(DOCX 13 kb) [file 40695_2016_18_MOESM1_ESM.docx]

**Changes to assay techniques across two decades of the Women’s Healthy Ageing Project**

Cholesterol

Total cholesterol, HDL cholesterol, and triglycerides were meaured using standard methods on routine automated chemistry systems. For cholesterol the instrument total coefficient of variation of ≤3% and an assay range of 0.161-18.26 mmol/L. For triglycerides the instrument total coeffiecient of variation is ≤5%, with an assay range of 0.071-16.05 mmol/L. LDL cholestrol was calculated using the Friedwald formula adapted for SI units [1].

Glucose

Glucose was measured in plasma using standard methods on routine automated chemistry systems. The the instrument total coeffiecient of variation is ≤5%, with an assay range of 0.28-44.4 mol/L.

Insulin

Insulin was measured by radioimmunoassay using standard methods on routine automated chemistry systems. The instrument total coeffiecient of variation is ≤7%, with an assay range of 1-300.0 μU/mL.

Iron studies

Iron studies were measured according to standard methods on routine automated chemistry systems. The total coefficient of variation for iron was ≤4.7% with an assay range of 0.97-318.4 μmol/L. For ferritin the total coefficient of variation for iron was ≤9%, with an assay range of 0-2000 ng/mL. The assay range for transferrin is 0.09-28.515 g/L, with a total coefficient of variation of ≤5%.

Homocysteine was measured using a commercial fluorescence polarisation assay developed by Abbott for their IMx Analyzer from 1991 to 1999 [2], and the DPC Immulite 2000 Analyser with DPC Reagents from 2002 to 2013. From 2013 the Abbott Architect with Architect Homocysteine Reagent Kit was used. The the instrument total coeffiecient of variation is ≤10%, with an assay range of 0-50.0 μmol/L.

Oestradiol, FSH, LH, and Inhibin

Hormones were measured by automated microparticulate enzyme immunoassay. The correlation coefficient was 0.99 and 0.98 for FSH and LH respectively. Inhibin had a coefficient of variation of 6.8% (Inhibin), Oestradiol assay had a sensitivity of 20pmol/L (Oestradiol) and inter-assay coefficient of variation 6.6% at 400 pmol/L, and between-assay coefficient of variation 11% at 110 pmol/L, 13% at 470 pmol/L and 13% at 1160 pmol/L.

SHBG and DHEAS

SHBG and DHEAS were measured by automated enzyme immunoassay. The intra- and inter-assay coefficients of variation for SHBG measures using this instrument were 3.2% and 11.3% respectively, and for DHEAS measures were 9.5% and 13% respectively.

Testosterone

Total serum testosterone was measured using double-antibody RIA, after sample sample extraction and polyethalene glycol-enhanced separation of bound from free ligand with ^125^Itestisterone (iodinated testosterone) as tracer. The between-assay coefficient of variation at a testosterone level of 2nmol/L was 5.8 to 12%. With an assay range of 0.35 – 52.1 nmol/L and a total coefficient of variation of ≤13.3%.

Vitamin D

Vitamin D was measured using standard methods. The instrument assay range is 0.0–160.0 ng/mL.

**References**

1. Guthrie JR, Ball M, Dudley EC, Garamszegi CV, Wahlqvist ML, Dennerstein L et al. Impaired fasting glycaemia in middle-aged women: a prospective study. International journal of obesity and related metabolic disorders : journal of the International Association for the Study of Obesity. 2001;25(5):646-51. doi:10.1038/sj.ijo.0801569.

2. Guthrie JR, Clark MS, Dennerstein L, Burger HG. Serum C-reactive protein and plasma homocysteine levels are associated with hormone therapy use and other factors: a population-based study of middle-aged Australian-born women. Climacteric : the journal of the International Menopause Society. 2005;8(3):263-70. doi:10.1080/13697130500162611.

3. Burger HG, Dudley EC, Hopper JL, Shelley JM, Green A, Smith A et al. The endocrinology of the menopausal transition: a cross-sectional study of a population-based sample. J Clin Endocrinol Metab. 1995;80(12):3537-45.
